# Brown Tumors: The Hidden Face of Primary and Renal Hyperparathyroidism Amid Real-Life Settings

**DOI:** 10.3390/jcm13133847

**Published:** 2024-06-29

**Authors:** Mara Carsote, Mihai-Lucian Ciobica, Oana-Claudia Sima, Ana Valea, Cosmina Ioana Bondor, Andreea Geleriu, Madalina Ticolea, Claudiu Nistor, Crina Claudia Rusu

**Affiliations:** 1 Department of Endocrinology, “Carol Davila” University of Medicine and Pharmacy, 050474 Bucharest, Romania; carsote_m@hotmail.com; 2 Department of Clinical Endocrinology V, C.I. Parhon National Institute of Endocrinology, 011863 Bucharest, Romania; oana-claudia.sima@drd.umfcd.ro; 3 Department of Internal Medicine and Gastroenterology, “Carol Davila” University of Medicine and Pharmacy, 020021 Bucharest, Romania; 4 Department of Internal Medicine I and Rheumatology, “Dr. Carol Davila” Central Military University Emergency Hospital, 010825 Bucharest, Romania; 5 PhD Doctoral School, “Carol Davila” University of Medicine and Pharmacy, 050474 Bucharest, Romania; 6 Department of Endocrinology, “Iuliu Hatieganu” University of Medicine and Pharmacy, 400012 Cluj-Napoca, Romania; 7 Department of Endocrinology, County Emergency Clinical Hospital, 400347 Cluj-Napoca, Romania; 8 Department of Medical Informatics and Biostatistics, “Iuliu Hatieganu” University of Medicine and Pharmacy, 400012 Cluj-Napoca, Romania; cbondor@umfcluj.ro; 9 Department of Endocrinology, Sibiu Clinical County Hospital, 550245 Sibiu, Romania; geleriu.andreea@yahoo.com; 10 Department of Pathophysiology, “Iuliu Hatieganu” University of Medicine and Pharmacy, 400012 Cluj-Napoca, Romania; madalinaticolea@gmail.com; 11 Department 4—Cardio-Thoracic Pathology, Thoracic Surgery II Discipline, “Carol Davila” University of Medicine and Pharmacy, 050474 Bucharest, Romania; 12 Thoracic Surgery Department, “Dr. Carol Davila” Central Emergency University Military Hospital, 010825 Bucharest, Romania; 13 Department of Nephrology, “Iuliu Hatieganu” University of Medicine and Pharmacy, 400012 Cluj-Napoca, Romania; claudia.rusu@umfcluj.ro; 14 “Mihai Manasia” Nephrology and Dialysis Clinic, County Emergency Clinical Hospital Cluj, 400347 Cluj-Napoca, Romania

**Keywords:** brown tumor, parathyroid, surgery, bone, PTH, calcium, scintigraphy, osteoclast, parathyroidectomy

## Abstract

Brown tumors, an exceptional bone complication of severe primary (PHP) or renal (secondary) hyperparathyroidism (RHP), are caused by long-standing, elevated parathormone (PTH)-induced osteoclast activation causing multinucleated giant cell conglomerates with hemosiderin deposits in addition to the local production of cytokines and growth factors. We aim to present an adult case series including two females displaying this complication as part of a multidisciplinary complex panel in high PTH-related ailments. The approach was different since they had distinct medical backgrounds and posed a wide area of challenges amid real-life settings, namely, a 38-year-old lady with PHP and long-term uncontrolled hypercalcemia (with a history of pregnancy-associated PHP, the removal of a cystic jaw tumor, as well as a family and personal positive diagnosis of polycystic kidney disease, probably a PHP-jaw tumor syndrome), as well as, a 26-year-old woman with congenital single kidney and chronic renal disease-associated RHP who was poorly controlled under dialysis and developed severe anemia and episodes of metabolic acidosis (including one presentation that required emergency hemodialysis and was complicated with convulsive seizures, followed by resuscitated respiratory arrest). Both subjects displayed a severe picture of PHP/RHP with PTH levels of >1000 pg/mL and >2000 pg/mL and elevated serum bone turnover markers. Additionally, they had multiple brown tumors at the level of the ribs and pelvis (asymptomatically) and the spine, skull, and pelvis (complicated with a spontaneous cervical fracture). As an endocrine approach, the control of the underlying parathyroid disease was provided via surgery in PHP (for the postparathyroidectomy hungry bone syndrome) via medical intervention (with vitamin D analogs) in RHP. Additionally, in this case, since the diagnosis was not clear, a multidisciplinary decision to perform a biopsy was taken (which proved inconclusive), and the resection of the skull tumor to confirm the histological traits. This series highlights the importance of addressing the entire multidisciplinary panel of co-morbidities for a better outcome in patients with PHP/RHP-related brown tumors. However, in the instance of real-life medicine, poor compliance and reduced adherence to recommendations might impair the overall health status. Thus, sometimes, a direct approach at the level of cystic lesion is taken into consideration; this stands for a narrow frame of decision, and it is a matter of personalized decision. As seen here, brown tumors represent the hidden face of PHP/RHP, primarily the complex and severe forms, and awareness is essential even in the modern era.

## 1. Introduction

Brown tumors represent an exceptional complication of severe primary or renal (secondary) hyperparathyroidism as part of bone disease, noting there is an excessive growth of osteoclast activity with a high bone turnover and degraded mineralization as the primary pathogenic background in this specific matter [1,2,3]. Brown tumors are benign lesions formed by multinucleated giant cells associated with local microfractures, as well as hemosiderin deposits, which explains the brown macroscopic appearance [4,5,6]. In addition to the osteitis fibrosa cystica, they stand for the most dramatic bone involvement in progressive uncontrolled PTH (parathormone) excess regardless of the etiologic type of PTH extra-production at the level of parathyroid cells [7,8,9]. Nowadays, their prevalence is low, affecting less than 0.5% of the cases diagnosed with primary hyperparathyroidism, and is reported in approximately 2% of subjects with secondary/renal hyperparathyroidism [10,11,12]. They are more commonly found in older adults, with a peak incidence within the sixth and seventh decades of life [13,14,15]. Women are more frequently affected; for example, a male-to-female ratio of 1:3 under the age of 30 years was reported in patients with chronic kidney disease and high PTH [16,17,18]. The most affected skeletal sites are the pelvis, ribs, clavicle, extremities, and jaw, either as solitary or multiple lesions in prior known parathyroid conditions or as the first manifestation of an abnormal PTH level [19,20,21].

The pathogenic traits of brown tumors include increased PTH secretion, the local production of cytokines and growth factors [22,23,24], as well as a large panel of general anomalies of mineral metabolism as seen in chronic kidney failures, such as hyperphosphatemia, hypocalcemia, vitamin D deficiency, lack of calcitriol production, intestinal malabsorption of calcium, reduction of vitamin D receptor activity, and disturbances of the calcium-sensitive receptors in the parathyroid glands [25,26,27]. The excessive production of secondary PTH in terms of the calcium–phosphorus imbalance leads to the activation of osteoclasts, which are responsible for bone resorption. Osteoclastogenesis starts with the differentiation of hematopoietic stem cells into monocytes. Two main molecules promote the differentiation and functioning of osteoclasts, namely macrophage colony-stimulating factor (M-CSF) and RANKL (receptor activator of nuclear factor kappa-B). M-CSF is secreted and binds to its receptor on the surface of osteoclast precursor cells, triggering intracellular signaling events with the proliferation and differentiation of osteoclast precursor cells into mature osteoclasts. Furthermore, M-CSF enhances the expression of other crucial factors required for osteoclast function, such as RANK and NFATc1 (nuclear factor of activated T cells 1). IL-1β (interleukine) has also been shown to activate osteoclasts in the presence of M-CSF and RANKL [28,29,30]. At the level of brown tumors, local calcium anomalies induce vascular fragility, while the massive destruction of red blood cells in these lesions causes the accumulation of hemosiderin (which appears as a brown pigment during histological examination) [31,32,33]. Regarding the role of angiotensin II in the production of brown tumors, most patients with chronic kidney disease, especially in the advanced stages, display an activated renin-angiotensin-aldosterone system (RAAS). Since angiotensin II may induce RANKL expression in osteoblasts, it could lead to osteoclast activation. The multinucleated cells of the monocyte/macrophage lineage can also be the target of angiotensin II, as they can induce osteoclast cell genesis [34,35,36].

While brown tumors represent a hidden face of the current presentation in primary and renal hyperparathyroidism, their presence might complicate the overall clinical picture and outcome; they represent the signature of a long-term, poorly controlled PTH-related bone ailment. However, their management varies on the matter of clinical picture and general health status, local clinical (compressive) effects, and co-morbidities. In most cases, the preferred approach is conservative (this does not exclude the decision of immediate parathyroid surgery), but sometimes, the differentiation between a primary or secondary bone malignancy requires a biopsy. Overall, surgical removal is decided on a matter of individual considerations across a multidisciplinary team, but a timely diagnosis and an adequate intervention (either medial or surgical) in order to control PTH excess and its associated mineral metabolism anomalies remains the most important strategy for the best prognosis [37,38,39,40].

### Objective

We aim to present a case series introducing particular clinical aspects across a multidisciplinary practice in two patients with unexpected complications of long-standing high PTH, namely brown tumors. While both subjects displayed these exceptional skeletal findings, the approach was different since they had distinct medical backgrounds and posed a wide range of challenges amid real-life medicine-associated issues.

The patients agreed to the presentation of their medical data while being hospitalized. The local ethical committee approved the retrospective collection of their medical history, as shown at the end of the paper. In the Discussion section, a brief literature overview is provided to highlight the specific particularities of these cases.

## 2. Brown Tumors in Primary Hyperparathyroidism

### 2.1. Admission

This was a 38-year-old female who was admitted for persistent hypercalcemia upon its detection amid primary health care. She complained of asthenia, nausea, non-specific bone pain, and muscle cramps for several months. A few days before her hospitalization, she received a 60 mg denosumab injection (via endocrine evaluation as an outpatient) after identifying a total serum calcium value of 11.9 mg/dL (normal range: 8.4–10.2 mg/dL).

### 2.2. Medical Background

Her medical history included two pregnancies two years apart complicated with preeclampsia, with her second pregnancy resulting in a premature birth. She continued to have high serum calcium values for a few years; the first abnormal assay was detected five years prior, which was 12.91 mg/dL (normal range: 8.6–10.3 mg/dL), but she declined further medical investigations or treatment. She developed associated polycystic kidney disease as well as kidney stones that required two surgical interventions during both of her pregnancies in association with recurrent urinary tract infections, mild arterial hypertension, and the surgical removal of a benign cystic jaw tumor a few months before her most recent admission. Her family medical history included both her mother and her maternal grandfather, who was diagnosed with polycystic renal disease. No genetic testing was conducted (neither in her family). She had normal menstruation.

### 2.3. Blood Panel: Hormonal and Biochemistry Assays

The mineral metabolism panel showed a high-normal total serum calcium value of 10 mg/dL (following recent denosumab administration), a low serum phosphorus value of 1.9 mg/dL (normal range: 2.3–4.7 mg/dL), a markedly increased serum PTH level of 1123 pg/mL (normal range: 15–65 pg/mL) and a decreased 25-hydroxyvitamin D (25OHD) level of 12.6 ng/mL (normal range: 30–100 ng/mL) with normal kidney function. These assessments were consistent with the diagnosis of primary hyperparathyroidism; moreover, a secondary component due to vitamin D deficiency may have been co-present (Table 1).

Moreover, the biochemistry panel revealed mildly elevated fasting glycemia (with normal glycated hemoglobin A1c) and decreased serum iron (no anemia was confirmed on admission but during the early follow-up period) (Table 2).

The serum bone formation markers were increased, and the resorption marker CrossLaps level was mildly suppressed (Table 3).

Additionally, the thyroid panel showed normal function, with a thyroid-stimulating hormone (TSH) level of 0.7 μUI/mL (normal range: 0.5–4.5 μUI/mL), a free levothyroxine (FT4) value of 10.8 pmol/L (normal range: 9–19 pmol/L), and a negative anti-thyroperoxidase antibody value of 0.52 UI/mL (normal range: 0–5.61 UI/mL).

### 2.4. Imagery Assessments

#### 2.4.1. Confirmation of the Parathyroid Tumor

**Figure 1:**
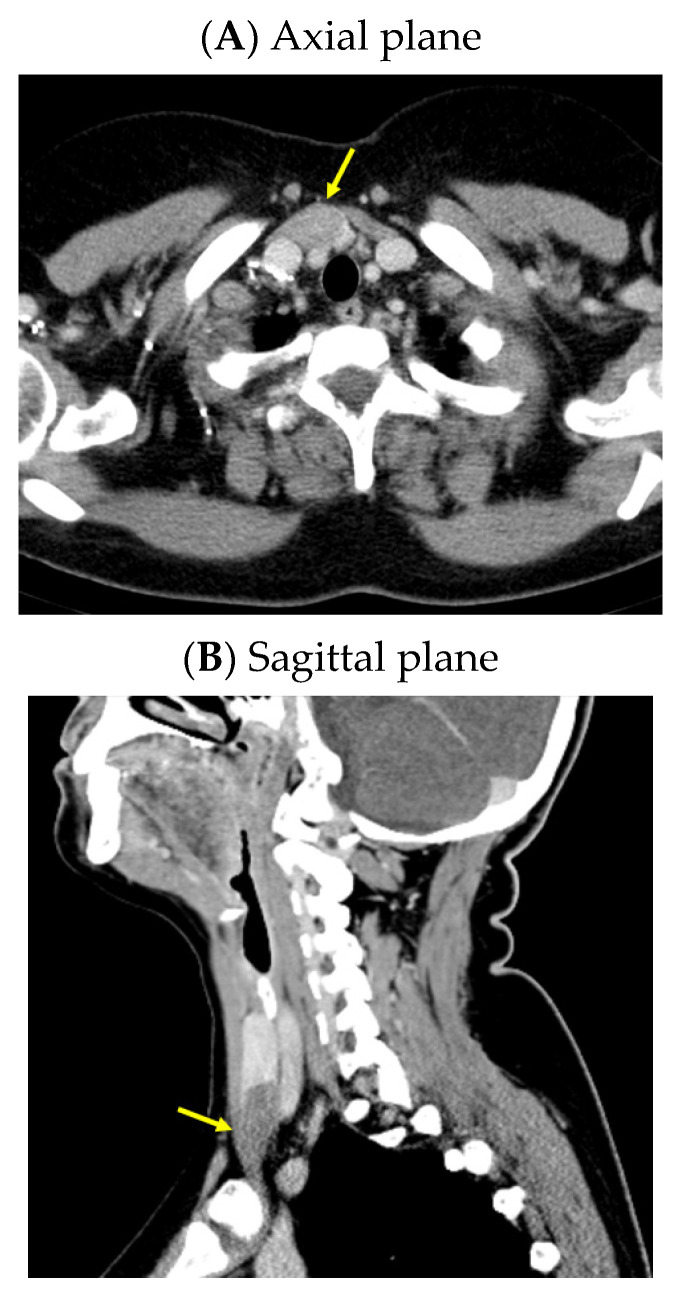

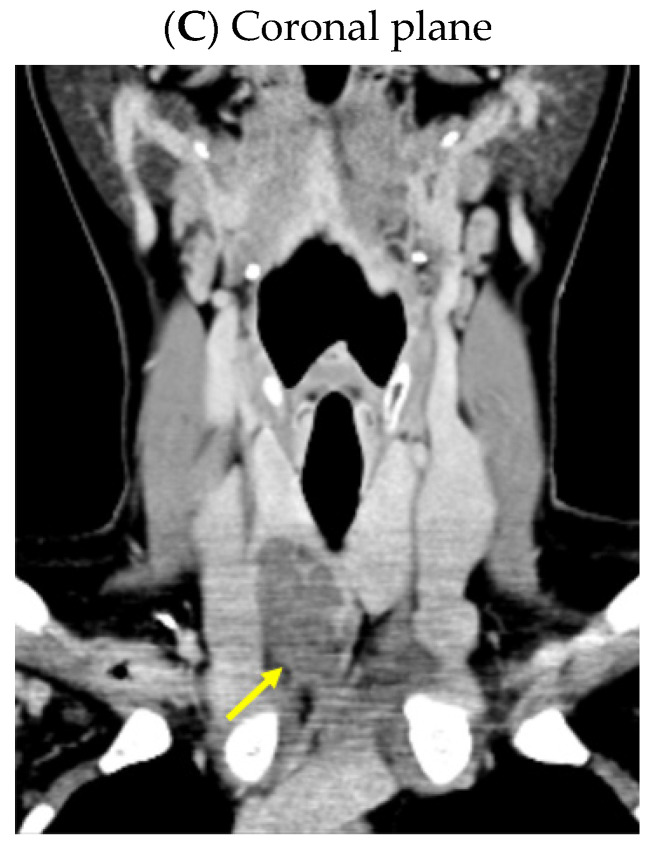
Intravenous contrast CT scan: right inferior parathyroid tumor (yellow arrow) displayed as a hypodense nodule of 3.98 by 1.13 by 2.53 cm.

An anterior neck ultrasound revealed that posterior and inferior to the right thyroid lobe, a hypoechoic, inhomogeneous, and highly vascularized nodule of 3.98 by 1.13 by 2.53 cm was found, suggestive of a right inferior parathyroid tumor. Furthermore, a right thyroid lobe of 1.70 by 1.36 by 5.25 cm and a left thyroid lobe of 1.60 by 1.12 by 5.11 cm with a hypoechoic, inhomogeneous, micronodular pattern and normal vascularization were identified; the isthmus was of 0.35 cm with a hypoechoic, inhomogeneous nodule of 1 by 0.50 by 0.97 cm. A computed tomography (CT) scan confirmed the single parathyroid tumor (a hypodense nodule of 2.48 by 1.80 by 3.69 cm) (Figure 1).

**Figure 2:**
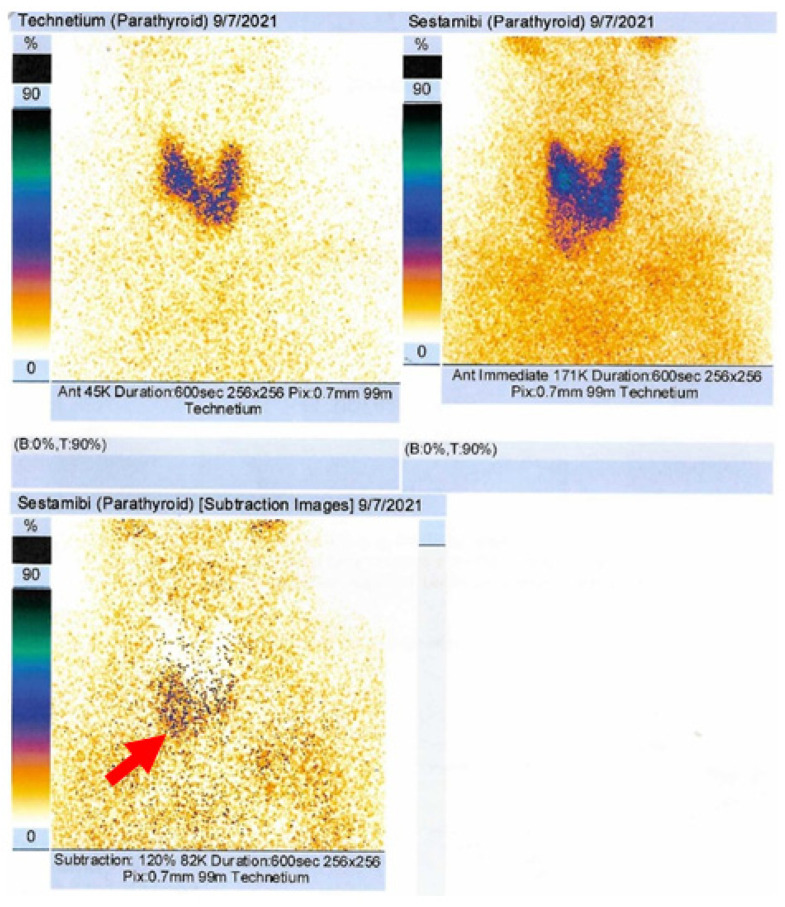
Dual-tracer 99m-Tc parathyroid scintigraphy with 99m-Tc pertechnetate (111 MBq) and 99m-Tc sestamibi (666 MBq; effective dose of 7.43 mSv; 120% subtraction captures at 10 min) show an area of late increase tracer uptake at the inferior to the lower half of the right thyroid lobe, suggesting a right inferior parathyroid tumor (red arrow).

Dual-tracer 99m-Technetium (99m-Tc) pertechnetate and sestamibi scintigraphy revealed a late increase uptake at the inferior to the lower half of the right thyroid lobe, consistent with a right inferior parathyroid tumor (Figure 2).

#### 2.4.2. Identification of the Brown Tumors

**Figure 3:**
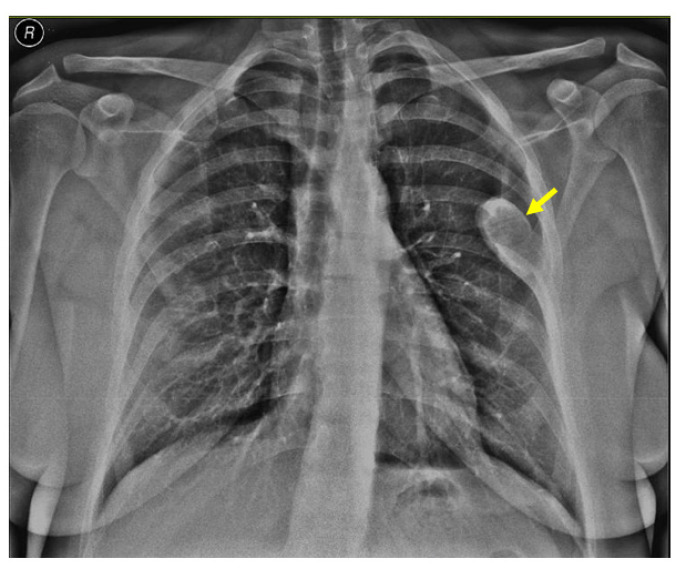
X-ray showing a brown tumor: a single, oval, well-shaped lesion of 6.4 by 2.7 cm in the lateral half of the left posterior sixth rib (yellow arrow).

Noting the bone pain on admission, an X-ray screening was performed and showed a rib tumor suggestive of a brown tumor (a solitary, oval, well-shaped tumor of 6.4 by 2.7 cm in the lateral half of the left posterior sixth rib) (Figure 3).

**Figure 4:**
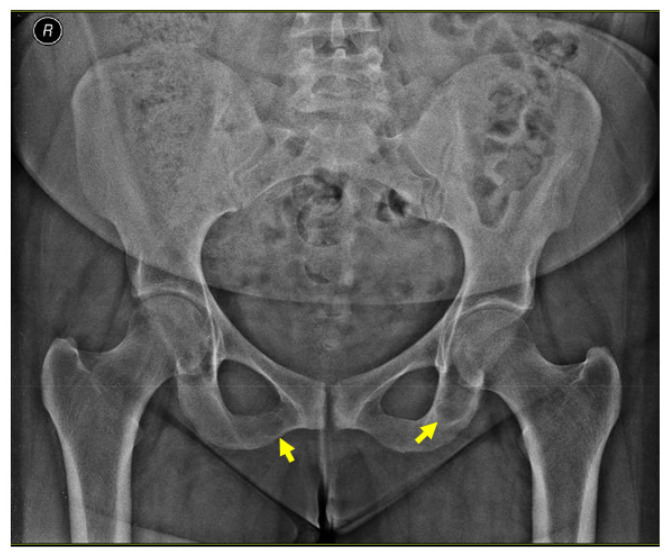
X-ray of the pelvis: brown tumors at the level of the ischium (yellow arrows).

An X-ray of the pelvis also revealed multiple osteolytic lesions of the ischium (brown tumor features) with normal femoral head sphericity and position and a mildly reduced coxo-femoral joint space (Figure 4).

**Figure 5:**
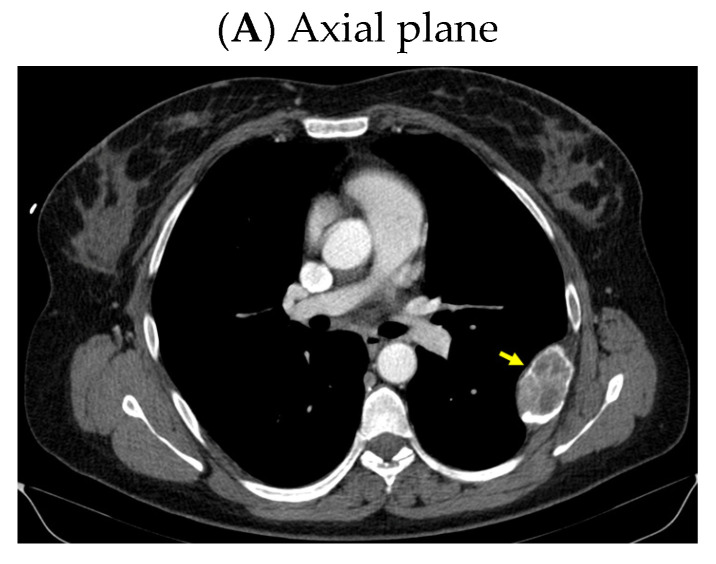

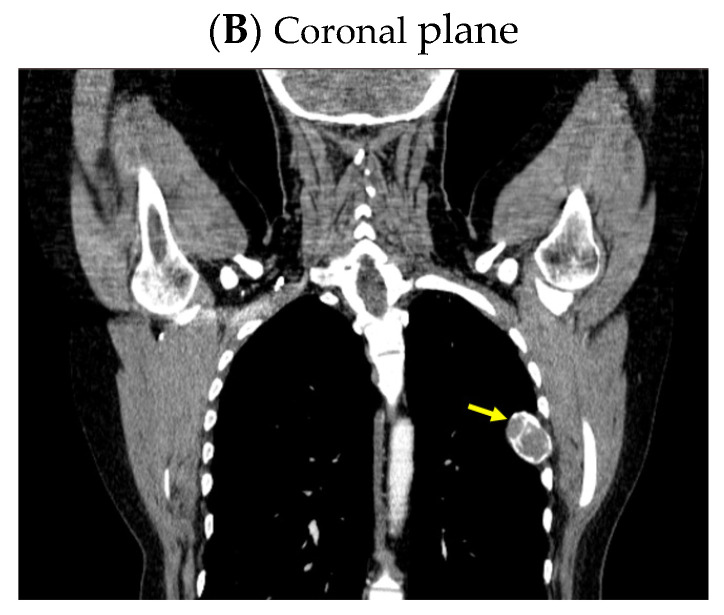
Intravenous contrast CT: a tumor mass located in the middle and posterior part of the left sixth rib, with median cortical destruction of 2.53 by 3.31 cm, suggestive of a brown tumor (yellow arrow).

A CT scan confirmed the rib tumor mass (highly suggestive of a brown tumor) located in the middle and posterior part of the left sixth rib that caused median cortical destruction (of 3.09 by 5.13 cm in the axial plane, 2.53 by 3.31 cm in the coronal plane reconstruction, and 6.11 by 3.46 cm in the sagittal plane reconstruction) (Figure 5).

**Figure 6:**
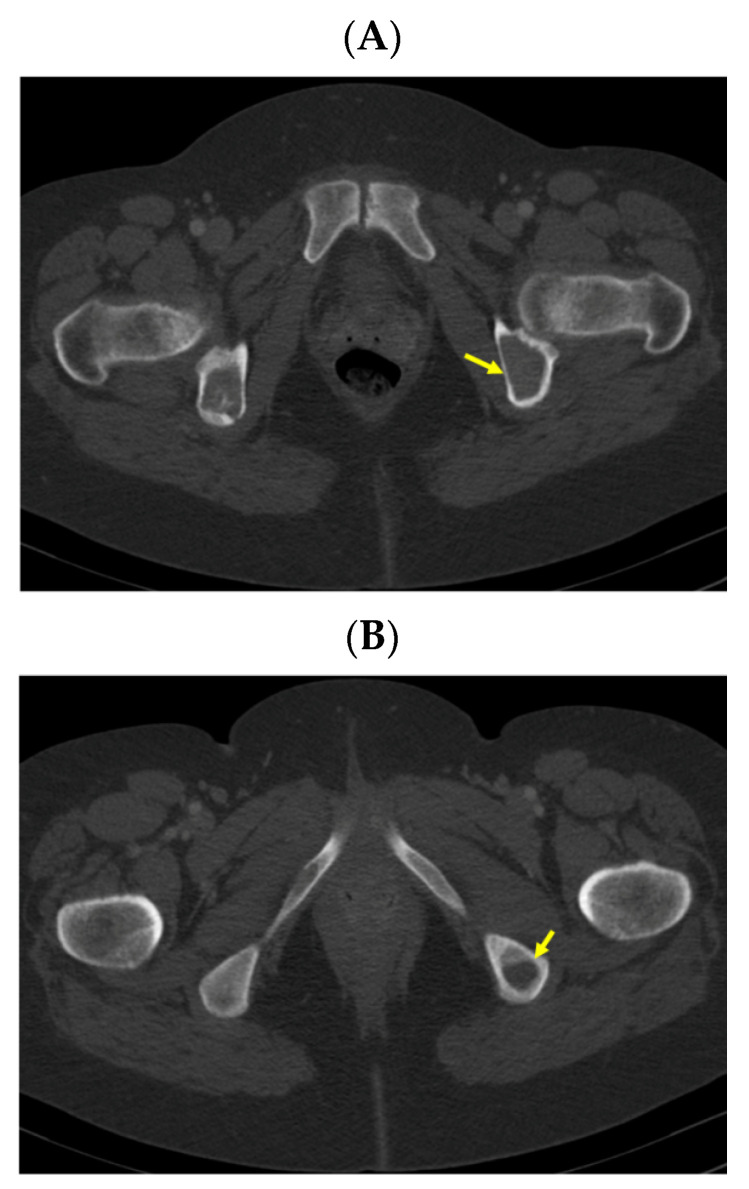
Pelvis CT scans showing other brown tumors: (**A**) osteolytic lesion of the left coxal bone adjacent to the acetabular fossa of 1.14 by 1.71 cm (axial plane); (**B**) osteolytic lesion of the left ischium of 1.97 by 3.29 cm (axial plane).

A pelvis CT scan confirmed the osteolytic lesions of the ischium and of the left coxal bone adjacent to the acetabular fossa, measuring 1.97 by 3.29 cm and 1.14 by 1.71 cm, respectively (brown tumors) (Figure 6).

**Figure 7:**
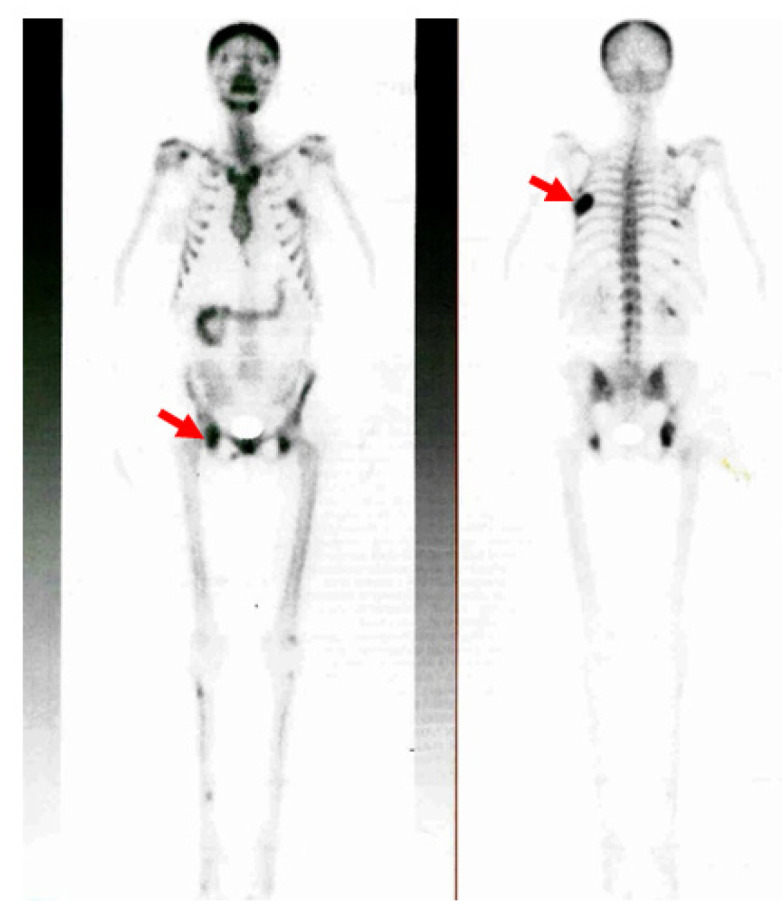
99mTc-HDP whole-body bone scintigraphy: locally increased uptake at the level of the sixth left rib (brown tumor), as well as of the seventh, ninth, and eleventh right posterior ribs, pelvis, and acetabular rim; moderately increased uptake at the scapula-humeral joints and the mandible; diffusely increased uptake at the skull (the red arrows show the brown tumors).

Additionally, the 99m-Tc hydroxymethylene diphosphonate (99mTc-HDP) whole-body bone scintigraphy showed a diffuse increased uptake at the level of the skull and at the local level in the posterior part of the sixth left rib (brown tumor); other mildly increased uptake areas were in the seventh, ninth, and eleventh right posterior ribs, pelvis, and acetabular rim (Figure 7).

### 2.5. Other Investigations

Other complications, such as low bone mineral density (BMD) for age and degraded bone microarchitecture, were confirmed. Specifically, a central dual-energy X-ray absorptiometry (DXA) evaluation (via a GE Lunar Prodigy device) showed a lumbar L1-L4 BMD of 1.006 g/sqcm with a Z-score of −2.3 SD, a femoral neck BMD of 0.915 g/sqcm with a Z-score of −1 SD, a total hip BMD of 0.979 g/sqcm with a Z-score of −0.6 SD, and a third distal radius BMD of 0.587 g/sqcm with a Z-score of −1.8 SD. The trabecular bone score (TBS) was 0.967.

**Figure 8:**
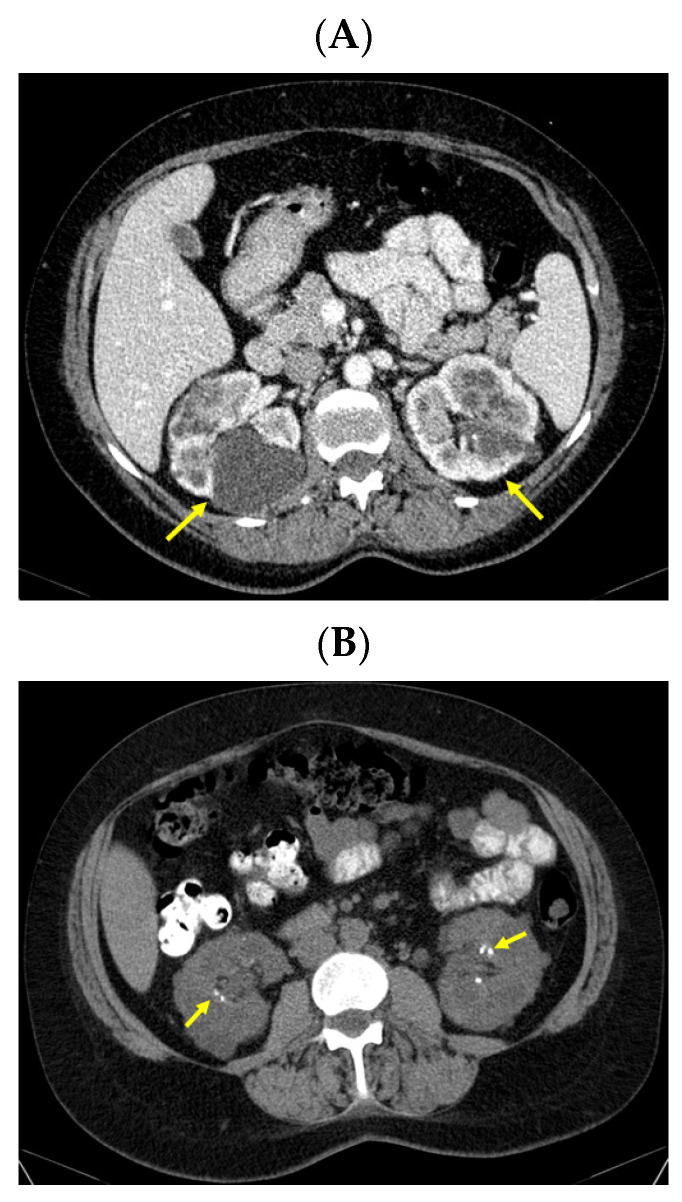
Intravenous contrast abdominal CT showing: (**A**) both kidneys with multiple cystic lesions both located in and protruding from the renal cortex bilaterally, the posterior part of the right kidney with a cyst of 6.27 by 5.36 by 6.01 cm with mass effect on the phyllo-caliceal system and protruding from the kidney (the yellow arrows show cystic kidney disease) and (**B**) bilateral renal microstones in primary hyperparathyroidism in yellow (axial plane).

Moreover, an abdominal CT confirmed the bilateral polycystic features of the kidneys (with a convex contour due to multiple cystic lesions that were located in and protruding from the renal cortex; the posterior part of the right kidney contained the largest cyst of 6.27 by 5.36 by 6.01 cm with mass effect on the phyllo-caliceal system and protruding from the kidney). Of note, renal microlithiasis was co-present in both kidneys (Figure 8).

### 2.6. Management

Based on this evaluation, primary hyperparathyroidism-related hypercalcemia was confirmed; the cause was suggested to be a right inferior parathyroid tumor as revealed by the triple imagery assessments (neck ultrasound, CT scan, and Tc-based parathyroid scintigraphy). Other unexpected and prior undiagnosed complications were multiple brown tumors, while the adult lady had an intact kidney function with a familial polycystic kidney condition. She most probably had a history of high PTH and calcium amid a previous pregnancy. She proved extremely non-compliant with the medical and surgical recommendations over the years. Taking into consideration the young age at primary hyperparathyroidism identification in addition to the cystic renal condition (including in two other family members) and the prior diagnosis of a jaw tumor (with benign cystic traits), a genetic condition in terms of hyperparathyroidism-jaw syndrome was suspected, but further genetic testing (specifically the *CDC73* gene) was declined by the patient. Other endocrine assessments, such as a 24-hour serum calcitonin plasma metanephrines and normetanephrine assessment, were conducted, and the results were within the normal levels. Thus, a clinical diagnosis of multiple endocrine neoplasia type 2 was excluded.

**Figure 9:**
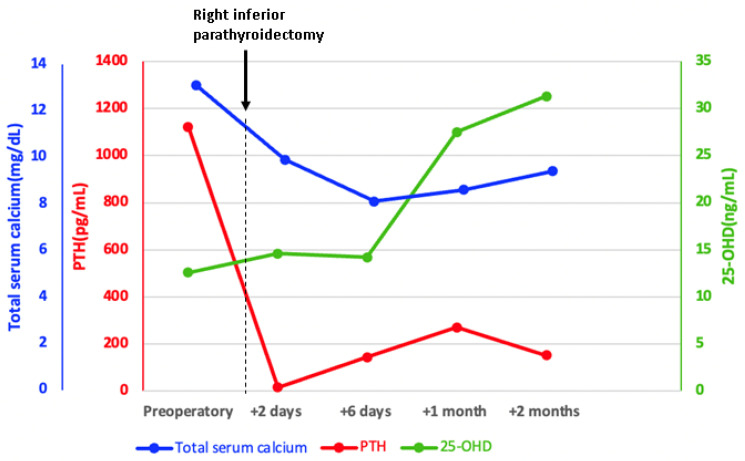
Evolution of total serum calcium, PTH (parathormone), and 25OHD (25-hydroxyvitamin D) following right inferior parathyroidectomy.

She underwent a right inferior parathyroidectomy with synchronous right thyroid lobe removal. Two days following parathyroidectomy, the total serum calcium and PTH decreased to normal levels. However, six days after parathyroid surgery, she developed a short episode of symptomatic hypocalcemia amid postoperative hungry bone syndrome that required prompt treatment with calcitriol 0.75–1 μg per day and intravenous calcium (1–2 g/day) for several days associated with an increased dose of cholecalciferol (4000 IU/day) and followed by oral calcium supplements (1.5 g/day) (Table 1). A good clinical outcome was registered, and the patient was offered to continue oral replacements. Within the following months, PTH became mildly increased, and this was regarded as secondary hyperparathyroidism due to incompletely corrected vitamin D supplementation followed by a mild deterioration of renal function. At the same time, no other parathyroid tumor was suspected (Figure 9).

**Figure 10:**
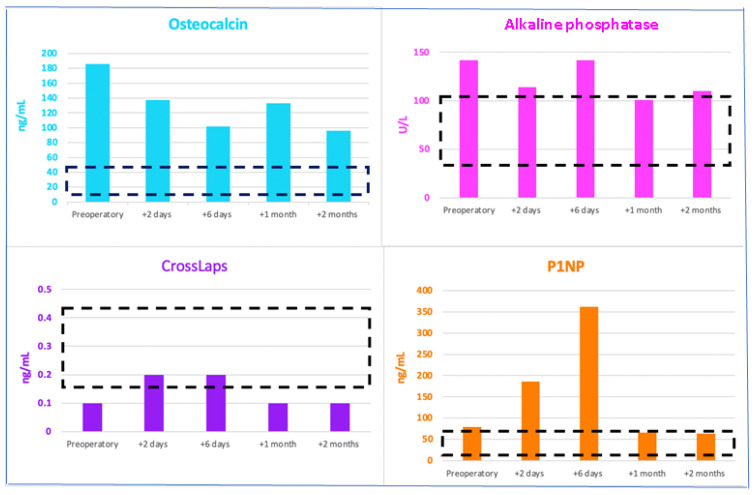
Profile of the serum bone formation markers (alkaline phosphatase, P1NP, and osteocalcin) and resorption marker CrossLaps before parathyroidectomy (but following a single 60 mg injection of denosumab) and during the first two months after surgery (the red arrow represents the timing of the right inferior parathyroidectomy and the black boxes represent the normal serum ranges of each parameter).

Of note, the bone formation markers osteocalcin and alkaline phosphatase decreased but remained above the normal range, while P1NP was higher than the preoperative level as a direct effect of the PTH level improvement (and part of the hungry bone syndrome); CrossLaps slightly increased and then reached normal values (Figure 10).

The postoperative histological report revealed a giant parathyroid adenoma of 2.9 by 2.6 cm (weighing 8.6 g) with oxyphil cells and cellular polyploidy that was in contact with the lower pole of the right thyroid lobe, presenting a smooth, discontinuous capsule without vascular invasion. The parathyroid tumor cells had a lobular pattern with round or oval nuclei, isolated mitoses, and areas of hyalinization (no vascular or perineural invasion was detected nor the areas of necrosis). A fragment of the adjacent thyroid parenchyma was present, with a follicular pattern, cuboidal and squamous epithelia, and free peripheral blood vessels (a follicular adenoma without any malignancy traits). The immunohistochemistry analysis of the parathyroid adenoma showed positive parafibromin/CDC73 status in relatively frequent tumor cells, as well as positive chromogranin A and Bcl2 in the tumor cells with a p53 of 3% in the wild-type tumor cells and a Ki67 proliferation marker of 4%.

### 2.7. Outcome

**Figure 11:**
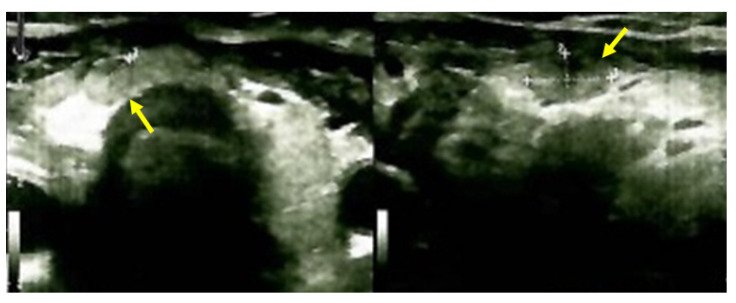
Neck ultrasound after right subtotal thyroidectomy and right inferior parathyroidectomy: an isoechoic and relatively homogeneous thyroid pattern with an intensely vascularized, inhomogeneous nodule of 0.91 by 0.54 by 0.78 cm (yellow arrow) at the right lobe-isthmus junction, which required follow-up; the images did not suggest another parathyroid tumor was present.

A clinical improvement was registered early after parathyroidectomy with the remission of the presurgery bone complains. The lady was offered therapy with calcitriol 0.25 μg/day, cholecalciferol 2000 UI/day, and oral calcium intake. No thyroxine replacement was necessary. No other postoperative complications were registered. No specific intervention was considered for the brown tumors other than managing the underlying parathyroid/mineral metabolism status according to a lifelong surveillance under these specific circumstances (Figure 11).

## 3. Brown Tumors in Renal Hyperparathyroidism

### 3.1. Admission

This was a 26-year-old female diagnosed with osteolytic lesions of the entire skeleton (brown tumors) caused by secondary (renal) hyperparathyroidism amid long-standing poor compliance to chronic dialysis. The young woman was first admitted as an emergency caused by the alteration of her general status due to severe kidney failure complicated with metabolic acidosis. She required hemodialysis in an emergency. During the third dialysis session, the patient developed convulsive seizures, followed by resuscitated respiratory arrest.

### 3.2. Medical History

The lady was known to have chronic kidney disease caused by a congenital single kidney associated with reflux nephropathy and secondary chronic tubule-interstitial lesions. She was not under any nephrology surveillance and showed poor adherence to primary health care check-ups.

### 3.3. Investigations amid Newly Detected Seizures during Dialysis

The laboratory data of that moment were consistent with the identification of hypocalcemia and secondary/renal hyperparathyroidism with extremely elevated PTH (but with normal serum phosphorus) associated with a high bone turnover, as reflected by increased alkaline phosphatase and severe anemia (Table 4).

Additionally, a brain CT was performed in order to highlight any organic traits as a cause of the mental status alteration, and an osteo-fibrous tumor formation was identified at the level of the left nasal fossa.

### 3.4. Management

Considering the severe hyperparathyroidism and associated damage to the mineral metabolism, paricalcitol (1 mg/day) was administered according to the nephrology guidelines (associated with daily 0.5 µg calcitriol). For the left nasal fossa tumor, an otorhinolaryngology assessment was conducted; a bone biopsy was recommended and performed. However, the histological report was inconclusive. The patient was stabilized and her treatment included a chronic dialysis program at an external nephrology center.

### 3.5. Outcome

**Figure 12:**
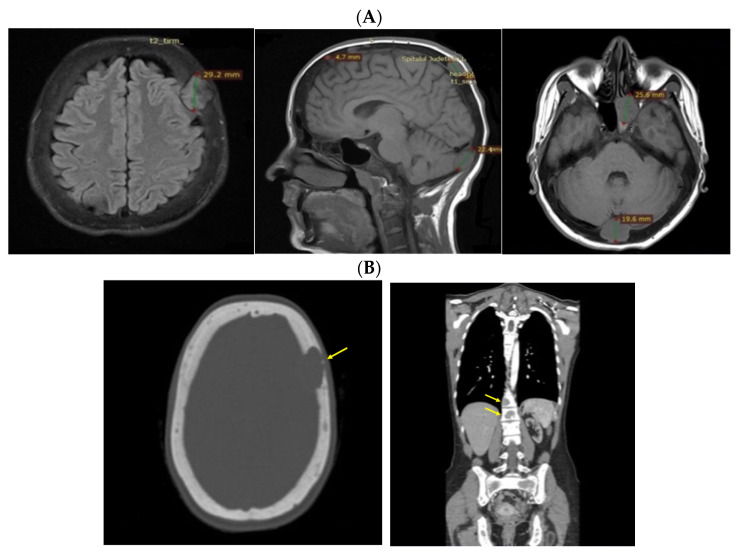
Brown tumors in an adult lady with poorly controlled renal hyperparathyroidism. (**A**) Native craniocerebral magnetic resonance imagery (the lesions are highlighted as well as their largest diameter). (**B**) Intravenous contrast computed tomography showing the mentioned lesions (yellow arrow) at the level of the skull (**left**) and spine (**right**).

Despite recommendations, she proved poorly compliant once again, and she was re-admitted only seven months later when she suffered a spontaneous fracture at the second cervical vertebra that required immobilization with a cervical collar. Amid this novel hospitalization, the PTH levels remained high with a reduced alkaline phosphatase level (Table 4). A native spine CT revealed an odontoid fracture involving the C1 anterior arch, also confirmed with a native magnetic resonance imaging (MRI) evaluation. Moreover, other left intramaxillary bone lesions were discovered. In this context, the examinations were extended to the level of the skeleton, and the head-thorax-abdomen-pelvis CT scan with intravenous contrast revealed multiple lesions located at different levels, such as the skull (of 3.5 by 3.8 cm), sternum (of 2.9 cm), sacrum (of 4 by 5.5 cm), and pelvis (of 6.5 by 5.5 cm) (Figure 12).

### 3.6. Approach of the Skull Tumors

**Figure 13:**
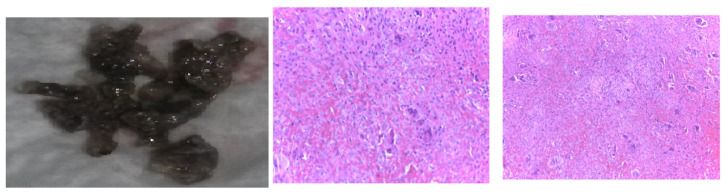
Brown tumor of the skull in chronic renal disease-related severe hyperparathyroidism: typical color features at the macroscopic level that stands for the tumor’s name (**left**); Both center and right subfigures are microscopic exams: hematoxylin-eosin (X10).

The multidisciplinary decision (also including a neurosurgical and neurological evaluation and assessment of the tumor-associated risks and the need for a clear distinction from oncologic traits) meant that a skull tumor lesion was enucleated. The histological report showed, at the macroscopic level, brown features with a crumbly consistency and an overall apparently well-vascularized tumor. The histological exam revealed osteoclast-like multinucleated giant cells arranged on a background of fusiform cells and focal fibrosis, diffuse hemorrhage, various fragments with short and thin cancellous bone lamellae with osteoblastic activity, and areas of multiple osteoclast-related bone lysis. These lesions were consistent with the diagnosis of a brown tumor of the bone, while a malignancy was ruled out (Figure 13).

The treatment for secondary hyperparathyroidism with vitamin D analogs was continued, as mentioned above. Interestingly, the serum phosphorus value was normal towards the lower normal range. Parathyroidectomy was discussed in the case of unfavorable evolution but ruled out for this point.

## 4. Discussion

Across this two-patient series, we shared a multidisciplinary experience amid a complicated panel of primary (probably genetic) hyperparathyroidism and renal hyperparathyroidism in a patient undergoing dialysis. Both ladies presented severe forms of parathyroid conditions with extremely elevated PTH values on first admission and had poor compliance with the recommendations over the years. The first case had brown tumors of the bone at the ribs and pelvis, while the second case had brown tumors at the skull, spine, and pelvis. The approach of the underlying parathyroid condition was distinct: prompt parathyroidectomy was performed in the case of primary hyperparathyroidism with a good clinical outcome (other than a short episode of hungry bone syndrome), while for the subject with chronic renal disease, vitamin D analogs were chosen at that time (the patient declined surgery, which had a high risk due to the severe alteration of their general health status, and they were poorly adherent to nephrology surveillance). Moreover, a specific intervention at the level of brown tumors was conducted in terms of biopsy and even removal since a prompt histological confirmation of the non-malignant features was mandatory (once the control of the underlying parathyroid condition was less likely to be achieved very soon in order to confirm the brown tumor regression). A brief literature overview pinpoints several key messages of these cases as follows.

### 4.1. Poor Compliance/Adherence to Recommendations and Severe Parathyroid Conditions

Both females had a long history of hypercalcemia and chronic renal disease related to a congenital kidney malformation. While further investigations were declined in the case of primary hyperparathyroidism until the most recent admission that finally led to parathyroid surgery, in the case of the woman with a prior renal congenital condition, the lack of adherence to long-term dialysis and vitamin D analog therapy caused a complicated picture of secondary (also named tertiary) hyperparathyroidism [41,42,43]. Of note, a component of secondary hyperparathyroidism (induced by low 25OHD) was identified in the first case as a potential (additional) contributor to the early postoperative PTH increase and postparathyroidectomy hungry bone syndrome (another secondary component was suggested by mild kidney dysfunction that was noted a few months after parathyroid surgery) [44,45,46]. Additionally, generally, poor adherence to traditional medical and surgical recommendations was noted amid the recent COVID-19 pandemic, as similarly seen in other multidisciplinary domains [47,48,49,50]. Overall, the clinical picture of primary/renal hyperparathyroidism included, in both cases, brown tumors that stood for a severe parathyroid ailment with a long history of uncontrolled PTH levels.

### 4.2. Brown Tumors and Highly Elevated PTH

The positive and differential diagnosis of brown tumors remains a challenge even in the modern era. Sometimes, they are mistaken as malignant lesions and mimic the pathological features of giant cell tumors at the bone level (as seen in our second case) [51,52]. Others remain completely asymptomatic and may be accidentally identified during investigations for hyperparathyroidism, like our subject with a primary type of PTH increase [53,54]. Generally, brown tumors, a consequence of the PTH-associated abnormal bone repair process, represent the hidden (or forgotten) face of primary/renal hyperparathyroidism or a traditional complication belonging to the bone panel in historical (traditional) forms of severe PTH-dependent conditions that might be associated with multiple other synchronous digestive, neurologic, cardiologic, and renal complications [55,56,57]. Nowadays, the facelift of the primary hyperparathyroidism presentation includes asymptomatic or normoglycemic forms without any complications or associating only with a limited area of mild symptoms, and this aspect is due to early detection and prompt screening calcium protocols in the general population followed by an adequate and efficient intervention to control high PTH and calcium levels (if any) [58,59,60]. Nevertheless, this was not our case in this instance.

Brown tumors, regardless of whether symptomatic or not, represent the signature of severe primary/renal hyperparathyroidism. Serum levels of PTH, calcium, phosphorus, and even tissue biopsies in selected cases are beneficial in confirming this conundrum. These tumors may imply important clinical consequences, such as bone pain, fractures, local deformities, neurologic consequences (for example, epilepsy), breathing disturbances (in cases with rib cage involvement), functional impairment, and overall reduced quality of life. Therefore, early diagnoses are important for initiating an appropriate treatment and preventing further complications [61,62,63]. In the first case, the surgical approach of the parathyroid tumor was the therapy of choice (following a single denosumab injection in order to control hypercalcemia) since the brown tumors displayed a mildly symptomatic picture in terms of non-specific bone pain (which otherwise may have been a consequence of extremely high PTH effects regardless of the presence of these bone tumors). Furthermore, brown tumor regression is expected, and radiologic surveillance is mandatory in addition to an adequate vitamin D replacement. In the second case, treatment with paricalcitol, an analog of vitamin D, and calcitriol, without restrictions related to the values of serum calcium and phosphorus, was offered to the lady. Moreover, bisphosphonates and denosumab are potential pharmacological alternatives under these circumstances, as well as calcimimetics or parathyroidectomy [64,65].

### 4.3. Bone Turnover Markers: A Friendly Tool for Practitioners

Serum alkaline phosphatase (which reduces the extracellular concentration of pyrophosphate, which is an inhibitor of mineralization) in patients with PTH-related brown tumors is usually very elevated, as seen in both cases [66,67]. In primary and renal hyperparathyroidism, its high level might correlate with increased preoperative PTH, low serum calcium, more extended surgery hospitalization amid parathyroidectomy, and a higher rate of postsurgery hungry bone syndrome (as seen in the first subject) [68,69]. Additionally, the bone turnover marker profile is expected to improve under PTH level normalization/improvement [70,71]. Furthermore, the surveillance of anti-osteoporotic medication might benefit from the marker profile assessment as seen in other types of primary and secondary osteoporosis [72,73]. A supplementary contributor to high alkaline phosphate is represented by severe vitamin D deficiency [74,75].

### 4.4. Imagery Assessment of Brown Tumors

Based on the radiological appearance, brown tumors can be mistakenly considered a metastatic disease (for instance, from primary malignancies such as breast, renal, and even thyroid carcinoma) or other bone metabolic/rheumatologic conditions. Apart from the traditional features identified on an X-ray and CT scan, these lytic lesions are assessed using a traditional whole-body bone scan (as seen in our first case) or PET/CT (which was performed in the second case, but the captures are not available; the results were not prone for malignancy in this instance). Various tracers are used in positronic emission tomography/computed tomography (PET/CT), such as [^18^F]-fluorodeoxyglucose, [^18^F]-fluorocholine, [^11^C]-fluorocholine, [^18^F]-sodium fluoride, [^68^Ga]-DOTATATE, and [^11^C]-methionine. Recent data has suggested that [^18^F]-fluorocholine PET/CT might be the most adequate tracer in brown tumors in order to provide a distinction from malignant tumors. Of note, after PTH excess is surgically or medically controlled, imagistic re-evaluation proving tumor regression is conclusive for a non-malignant lesion [76,77,78,79,80].

### 4.5. Decision of Biopsy and Resection for Brown Tumors: A Personalized Matter

Generally, different types of bone biopsies might help the overall management if a malignancy at the bone level is suspected, particularly in patients who are already recognized with a prior cancer of any origin [81,82,83,84]. Nevertheless, in brown tumors, the choice of performing a biopsy stands on an individual matter due to the immediate importance of having a histological report (as seen in our second case). The procedure might not be conclusive, and the main decision factors remain the management of the underlying parathyroid condition and associated general and local ailments. In these cases, close surveillance is mandatory and, if necessary, repeating the procedure or having a tumor enucleation/resection only in a limited number of cases (particularly in those where the clinical, hormonal, and imaging-based diagnosis and follow-up are not highly suggestive of brown tumors, and lesion remission was not noted) [85,86,87]. Notably, in the second case, the decision was multidisciplinary at that point, and this stands for real-life settings. Alternatively, brown tumor detection following the correction of primary hyperparathyroidism but still causing local complications, such as spinal cord compression and myelopathy, may be referred to a neurosurgical intervention [88]. Mostly, the decision of surgery depends on the local compressive effects and esthetic/functional purposes, particularly in renal hyperparathyroidism, which displays a more severe picture than the primary type and a lower rate of controlled PTH values [89,90]. For instance, we mention a case of a 53-year-old female with secondary hyperparathyroidism amid a six-year history of dialysis who developed brown tumors at the level of the maxilla and mandible, and incisional biopsy was required in order to confirm the histological profile; this was followed by parathyroidectomy and PTH profile improvement without the rapid regression of the bone lesion causing a functional impairment; thus a tumor excision (mandibular osteotomy) was taken into consideration [89]. Similarly, a 37-year-old male with a 15-year history of end-stage kidney disease was confirmed with a large tumor at the level of the sphenoid wing, which required resection via craniotomy due to local effects [90].

### 4.6. Other Aspects of Primary/Secondary Hyperparathyroidism Complicated with Brown Tumors

One issue to be mentioned is the general health status deterioration in the second case amid the presence of metabolic acidosis, seizures, cardiac arrest, and anemia in complicated chronic kidney disease, including severe secondary hyperparathyroidism. Some data have shown a correlation between metabolic biomarkers and bone profiles under similar circumstances [91,92,93]. Anemia represents a marker of long-standing disease that has been reported in patients with parathyroid tumors, including primary hyperparathyroidism, and it usually serves as a marker of a more deteriorated status and long-term, uncontrolled PTH excess; for instance, one study from 2022 showed a prevalence of 20% in subjects with intact kidney function [94].

**Figure 14:**
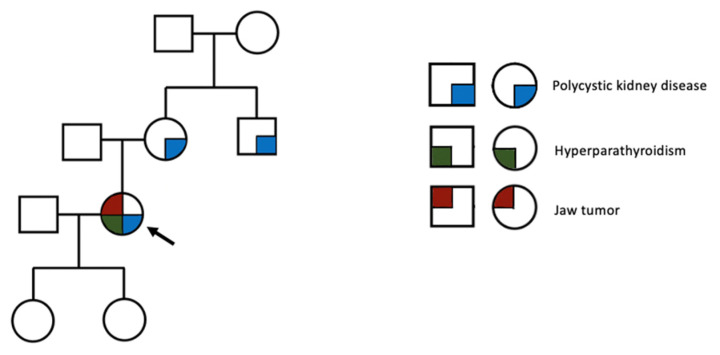
Family tree diagram of the index case (arrow): a 38-year-old female with primary hyperparathyroidism-related brown tumors (symbols: square: male; circle: female).

Additionally, in the first case, several practical points about the primary hyperparathyroidism profile should be mentioned, such as a long medical history of hypercalcemia amid two pregnancies. Yet, she proved poorly compliant with further medical exploration and treatment. During gestation, the clinical picture of the underlying parathyroid tumor was not specific, and it should have been differentiated from multiple other complications of the pregnancy. In contrast, the slightly reduced level of serum calcium during gestation might have masked hypercalcemia. Unrecognized and untreated hypercalcemia poses an increased maternofetal risk, including in the postpartum period (such as hypercalcemia crisis, pancreatitis, neonatal hypocalcemia, and the restriction of fetal growth) [95]. Moreover, she experienced hungry bone syndrome following parathyroidectomy; the indicators of such an episode (which is less frequently found than in renal hyperparathyroidism) are very high preoperative PTH and alkaline phosphatase levels, a larger parathyroid tumor, associated vitamin D deficiency, and even the presence of bone complications such as brown tumors [44,45,46]. Finally, no genetic testing was available for this patient, but the presence of a parathyroid tumor at a very young age, in addition to an early onset of polycystic kidney disease (with a positive family background) and a cystic jaw tumor, is highly suggestive of a hereditary condition, such as hyperparathyroidism-jaw syndrome (Figure 14).

The syndrome, with underlying *CDC73* pathogenic variants, is part of the hereditary forms of primary hyperparathyroidism (which stands for 10% of all cases); it is associated with a higher risk of parathyroid carcinoma (involving 15% of the parathyroid tumors), which may be diagnosed at a very young age. Notably, most (but not all) parathyroid tumors are parafibromin deficient, which was not our case. Lifelong surveillance is mandatory, as well as genetic confirmation, including of the family members (if found positive) [96,97,98,99,100].

## 5. Conclusions

**Figure 15:**
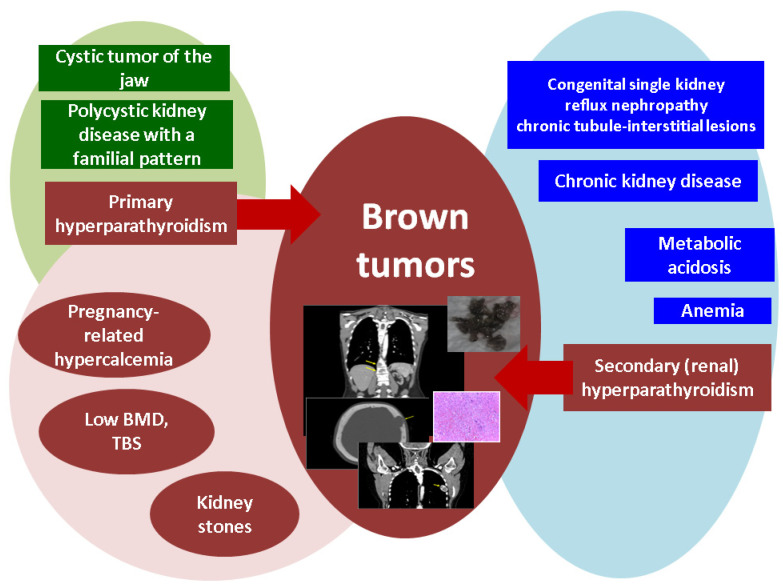
Sneak peek of the clinical picture in the cases with PTH-related brown tumors: on the left, there is the case with primary hyperparathyroidism; the green represents the components of the hyperparathyroidism-jaw tumor (including primary hyperparathyroidism), and the brown represents the elements of the primary hyperparathyroidism; on the right, there is the case with chronic kidney disease and associated complications (blue), including secondary (renal) hyperparathyroidism (abbreviations: BMD: bone mineral density; TBS: trabecular bone score).

Brown tumors represent the hidden face of primary and renal hyperparathyroidism, and awareness is essential even in the modern era. This case series highlights the importance of addressing the entire multidisciplinary panel of ailments in two young adult females who presented severe forms of parathyroid conditions associated with pregnancy-related hypercalcemia and hungry bone syndrome following parathyroidectomy; one case also displayed the features of a hyperparathyroidism-jaw tumor and multiple complications of poorly controlled chronic kidney disease, such as episodes of metabolic acidosis, severe anemia, seizures, and a deteriorated general health status. Since this is a real-life setting, it is important to stress the fact that both subjects were poorly adherent to medical checkups and therapy amid their medical history. Notably, the brown tumors were asymptomatic in one case, while in the other, a vertebral fracture was noted. As an endocrine approach, the control of the underlying parathyroid disease was provided via surgery in one case and a medical intervention in the renal patient. Additionally, in this case, since the diagnosis was not clear, a multidisciplinary decision was taken to perform a biopsy (which proved inconclusive), as well as the resection of the skull tumor in order to confirm the pathological traits. This specific approach belongs to a narrow frame of decision, and it stands for a personalized attitude (Figure 15).

## Figures and Tables

**Table 1:** Mineral metabolism amid hospitalization for primary hyperparathyroidism in a young adult female: on admission and following parathyroidectomy (PTH: parathormone; PT: parathyroid; 25OHD: 25-hydroxyvitamin D; * a few days after a subcutaneous injection of 60 mg denosumab).

Parameter	On Admission *	2 Days after PT Surgery	6 Days after PT Surgery	1 Month after PT Surgery	2 Months after PT Surgery	Normal Range
Total serum calcium (mg/dL)	10	9.7	**8.1**	8.6	9.4	8.4–10.2
Ionized calcium (mg/dL)	4.5	4.5	**3.9**	**3.7**	4	3.9–4.9
Total proteins (g/dL)	6.8	6.7	6	7.2	7.4	6.4–8.3
Phosphorus (mg/dL)	**1.9**	3.6	4.1	3	3.9	2.3–4.7
Serum creatinine (mg/dL)	1	1.1	1.2	1.1	1.05	0.5–1.2
Serum urea (mg/dL)	41.3	60	65	50.1	43	15–50
PTH (pg/mL)	1123 *	15.96	**144.1**	**271.4**	**151.9**	15–65
25OHD (ng/mL)	**12.6**	**14.6**	**14.2**	**27.5**	31.3	30–100

**Table 2:** Biochemistry panel of a 38-year-old female with persistent hypercalcemia amid the diagnosis of primary hyperparathyroidism (HDL: high-density lipoprotein; PT: parathyroid).

Parameter	On Admission	1 Month after PT Surgery	2 Months after PT Surgery	Normal Range
Uric acid (mg/dL)	5.1	4.1	3.6	2.6–6
Alanine aminotransferase (U/L)	18.1	15	11	0–31
Aspartate aminotransferase (U/L)	12.3	14	11	0–32
Total cholesterol (mg/dL)	199.2	196	**221**	0–200
HDL-cholesterol (mg/dL)	**39**	40.6	47	40–60
Triglycerides (mg/dL)	112	141	122	0–149
Fasting glycemia (mg/dL)	**110**	84.3	87	70–105
Glycated haemoglobin A1c (%)	**4.7**	5.5	NA	4.8–5.9
Sodium (mmol/L)	143	141	139	136–145
Potassium (mmol/L)	4.62	4.53	4.1	3.5–5.1
Magnesium (mg/dL)	1.84	1.81	1.7	1.6–2.6
Haemoglobin (g/dL)	12.1	**11.2**	**11.4**	12–15.5
Serum iron (μg/dL)	**47**	56.8	57	50–170

**Table 3:** Serum bone turnover markers in prolonged hypercalcemia amid primary hyperparathyroidism (P1NP: procollagen type 1 N-terminal propeptide).

Serum Bone Turnover Markers	On Admission	2 Days after Surgery	6 Days after Surgery	1 Month after Surgery	2 Months after Surgery	Normal Range
**Bone Formation Markers**						
Osteocalcin (ng/mL)	**185.6**	**137.7**	**101.8**	**132.7**	**95.9**	11–43
Alkaline phosphatase (U/L)	**142**	**114**	**142**	101	**110**	38–105
P1NP (ng/mL)	**79.23**	**186.1**	**362.1**	**65.6**	**63.7**	15.13–58.59
**Bone Resorption Marker**						
CrossLaps (ng/mL)	**0.1**	0.2	0.2	**0.1**	**0.1**	0.162–0.436

**Table 4:** Panel of the parameters for the admission of a young lady with chronic kidney failure-related renal/secondary hyperparathyroidism amid an emergency presentation of the alteration of her health status due to metabolic acidosis (PTH: parathormone).

Parameters	First Admission	7 Months Later	2 Years Later	Normal Values
Total serum calcium (mg/dL)	**7.3**	**8.4**	9.75	8.8–10.6
Phosphorus (mg/dL)	3.7	3.4	3.3	2.5–4.5
iPTH (pg/mL)	**2008**	**1605**	44	15–65
Alkaline phosphatase (U/L)	**1893**	**933**	**211**	30–120
pH	**7.1**	7.37	7.36	7.35–7.45
Bicarbonate level (mmol/L)	**12**	**17**	**18**	22–29
Hemoglobin (g/dL)	**6**	11.5	12	12–15.5
Albumin (g/dL)	**3.2**	3.7	4.1	3.5–5.2
**Recommended Therapy with Vitamin D Analogs**				
Paricalcitol (mg/day)	1	2	2	
Calcitriol (µg/day)	0.5			

## Data Availability

The research data that support the findings of this case series are not publicly available. Other medical records are available upon request in accordance with the hospital rules, patients’ consent, and the local ethics committee.

## Abbreviations

BMD	Bone mineral density
CT	Computed tomography
DXA	Dual-energy X-ray absorptiometry
HDP	Hydroxymethylene diphosphonate
25OHD	25-hydroxyvitamin D
FT4	Free levothyroxine
IL	Interleukine
M-CSF	Macrophage colony-stimulating factor
MRI	Magnetic resonance imagery
NFATc1	Nuclear factor of activated T cells 1
PTH	Parathormone
P1NP	Procollagen type 1 N-terminal propeptide
PET/CT	Positronic emission tomography/computed tomography
RANKL	Receptor activator of nuclear factor kappa-B
RAAS	Renin-angiotensin-aldosterone system
TSH	Thyroid-stimulating hormone
TBS	Trabecular bone score
Tc	Technetium
